# D-cycloserine to enhance extinction of cue-elicited craving for alcohol: a translational approach

**DOI:** 10.1038/tp.2015.41

**Published:** 2015-04-07

**Authors:** J MacKillop, L R Few, M K Stojek, C M Murphy, S F Malutinok, F T Johnson, S G Hofmann, J E McGeary, R M Swift, P M Monti

**Affiliations:** 1 Peter Boris Centre for Addictions Research, Department of Psychiatry and Behavioural Neurosciences, McMaster University/St. Joseph's Healthcare Hamilton, Hamilton, ON, Canada; 2 Homewood Research Institute, Homewood Health Centre, Guelph, ON, Canada; 3 Center for Alcohol and Addiction Studies, Brown University, Providence, RI, USA; 4 Department of Psychiatry, Washington University, St. Louis, MO, USA; 5 Department of Psychology, University of Georgia, Athens, GA, USA; 6 Department of Psychological and Brain Sciences, Boston University, Boston, MA, USA; 7 Providence Veterans Affairs Medical Center, Providence, RI, USA; 8 Division of Behavioral Genetics, Department of Psychiatry, Rhode Island Hospital, Providence, RI, USA

## Abstract

Cue-elicited craving for alcohol is well established but extinction-based treatment to extinguish this response has generated only modest positive outcomes in clinical trials. Basic and clinical research suggests that D-cycloserine (DCS) enhances extinction to fear cues under certain conditions. However, it remains unclear whether DCS would also accelerate extinction of cue-elicited craving for alcohol. The goal of the current study was to examine whether, compared with placebo (PBO), DCS enhanced extinction of cue-elicited craving among treatment-seeking individuals with alcohol use disorders (AUDs). Participants were administered DCS (50 mg) or PBO 1 h before an alcohol extinction paradigm in a simulated bar environment on two occasions. The extinction procedures occurred 1 week apart and were fully integrated into outpatient treatment. Subjective craving for alcohol was the primary variable of interest. Follow-up cue reactivity sessions were conducted 1 week and 3 weeks later to ascertain persisting DCS effects. Drinking outcomes and tolerability were also examined. DCS was associated with augmented reductions in alcohol craving to alcohol cues during the first extinction session and these effects persisted through all subsequent sessions, suggesting facilitation of extinction. Participants in the DCS condition reported significant short-term reductions in drinking, although these did not persist to follow-up, and found the medication highly tolerable. These findings provide evidence that DCS enhances extinction of cue-elicited craving for alcohol in individuals with AUDs in the context of outpatient treatment. The potential clinical utility of DCS is discussed, including methodological considerations and context-dependent learning.

## Introduction

One major etiological perspective on alcohol and other substance use disorders is that these are conditions of maladaptive learning, including molecular, cellular, operant and associative processes.^1, 2, 3, 4^ With regard to associative conditioning, there is extensive evidence from basic research suggesting that, over time, conditioned environmental stimuli have an important motivational role.^4, 5^ This is also based on human laboratory studies indicating that alcohol cues reliably elicit robust increases in subjective craving^6^ and, in turn, that craving typically predicts subsequent alcohol consumption.^7, 8^ Similarly, clinical studies have revealed that craving, in general and as a function of environmental cues, is associated with relapse.^9, 10^ Although not all studies support this link, suggesting that the relationship is more complex than simply cause and effect, craving is considered to have substantial importance in understanding addictive behavior.^11^

Accordingly, there has been interest in treatments to weaken conditioned associations between alcohol cues and cue-elicited cravings for alcohol. This approach is referred to as cue exposure treatment (CET) and is akin to exposure with response prevention approaches in the treatment of anxiety disorders.^12, 13^ Specifically, CET exposes patients to alcohol cues under controlled conditions with the prevention of their dominant response (that is, consumption). In doing so, the procedure uses extinction to reduce the contingency between alcohol cues and consumption, thereby decreasing cue-elicited craving in response to alcohol cues and, putatively, decreasing the probability of subsequent drinking. In terms of clinical outcomes, a meta-analysis of the existing trials of CET suggested limited efficacy,^14^ although significant heterogeneity of effect size was present, reflecting consistently negative outcomes for nicotine dependence and consistently positive outcomes for alcohol dependence.^15^

Pharmacological approaches may enhance the efficacy of CET and one particularly promising compound is D-cycloserine (D-4-amino-3-isoxazolidone; DCS), an antibiotic medication that is also a partial agonist at the glycine modulatory site at the glutamatergic *N*-methyl-D-aspartate (NMDA) receptor. NMDA receptors play a critical role in learning and memory^16, 17^ and DCS may enhance salutary new learning during therapeutic extinction. This possibility has been most extensively investigated in augmenting treatment for anxiety disorders. DCS has been found to enhance extinction-based treatment of specific phobia,^18^ obsessive-compulsive disorder,^19^ panic disorder^20^ and social anxiety disorder.^21, 22^ A recent meta-analysis suggested that, in aggregate, DCS enhances extinction-based treatment for anxiety disorders,^23^ although some more recent trials have not revealed significant effects.^24^

With regard to alcohol use disorders (AUDs), preclinical studies have suggested that DCS may enhance extinction to alcohol cues. Specifically, DCS has been shown to enhance extinction as evidenced by reduced reacquisition of alcohol-related conditioned place preference in mice^25^ and reduced priming-based reinstatement in rats.^26^ These findings are consistent with preclinical studies on other addictive drugs.^27^ However, positive effects on alcohol-related extinction have not been replicated in humans. In two small studies in which DCS was administered to non-treatment-seeking heavy drinkers, the first concluded that DCS (125 mg) had no significant effect on extinction to alcohol cues,^28^ and the second suggested that DCS (50 mg) resulted in an increase in craving during the first session relative to placebo (PBO).^29^ To date, the only investigation conducted in a clinical context was a small pilot study in which DCS (250 mg) did not have any significant effect when compared with PBO in alcohol-dependent individuals who had recently completed treatment, albeit with only five patients per condition.^30^ These findings are similar to the general literature on DCS and addictive behavior, where the preclinical literature is relatively robust but findings in human studies tend to be mixed.^31^ For example, one recent study found that DCS enhanced extinction of cue-elicited craving in nicotine-dependent individuals,^32^ whereas another trial with cocaine-dependent individuals revealed a more persistent increase in craving during the first exposure session in the DCS condition.^33^

The ambiguity in the literature may be attributable to variability in important methodological parameters for studying DCS.^31, 34, 35^ Unlike conventional pharmacotherapies that are dosed daily, several critical boundary conditions may be required to be met for DCS to enhance learning.^31, 34^ For example, high or chronic doses can cause DCS to act as a direct NMDA antagonist.^36, 37, 38^ Thus, a week between extinction periods and doses is recommended to maximize consolidation and to avoid receptor desensitization. The demonstration of initial cue-elicited craving, which is not present in all individuals,^39, 40^ is another important parameter as extinction cannot occur, much less be enhanced, if craving is not elicited in the first place. Finally, a last consideration appears to be treatment motivation. Analogue studies on DCS using non-treatment-seeking samples and *de novo* conditioning have yielded null findings,^41, 42^ suggesting that DCS effects are only present or are substantially more robust among clinically affected individuals.

With regard to AUDs, the studies to date have not conducted a strong test of DCS enhancement of extinction of cue-elicited craving, meeting the preceding boundary conditions. The primary goal of the current study was to address these existing limitations via a translational approach that integrated experimental human laboratory methods with active treatment in a clinical sample. Specifically, we examined whether two administrations of 50 mg DCS, 1 week apart, would accelerate the attenuation of craving in a validated extinction paradigm involving 70-min periods of multi-modal alcohol cue exposure with response prevention.^43, 44^ This protocol was implemented in individuals actively seeking treatment for AUDs immediately adjacent to treatment sessions. The primary hypothesis was that DCS would enhance extinction of cue-elicited craving for alcohol. In addition, for exploratory purposes, we also examined its effects on drinking outcomes and its tolerability.

## Materials and methods

### Participants

Participants were recruited from the community using advertisements. Inclusion criteria were: (1) presence of a DSM-IV AUD diagnosis (abuse or dependence); (2) heavy drinking (that is, ⩾14/7 drinks weekly, men/women^45^); (3) seeking alcohol treatment; (4) evidence of alcohol cue reactivity (that is, ⩾1 point increase in craving in response to alcohol cues compared with neutral cues presented in the laboratory); (5) age 21–65 years; (6) stable domicile and contact information; (7) completion of ninth grade education; and (8) medically appropriate according to physician examination. Exclusion criteria were: (1) history of severe alcohol withdrawal symptoms (that is, self-report of hospitalization, hallucinations or other symptoms of delirium tremens; or in-person evidence of significant withdrawal symptoms at the screening evaluation); (2) a history of epilepsy/seizures, renal problems or hepatic problems; (3) contraindicated medications (for example, ethionamide, isoniazid, selective serotonin reuptake inhibitors); (4) presence of other current Axis I psychiatric disorders, with the exception of nicotine dependence; (5) living with a participant in the study; (6) mandated to treatment; and (7) pregnant, nursing or seeking to conceive. In addition to the standard contraindicated medications, any other medications that the prospective participants were currently taking were reviewed by the study physician to determine possible negative interactions.

Thirty-seven participants met eligibility criteria and enrolled in the study. Participants were randomly assigned to either the DCS condition (*n*=19) or PBO condition (*n*=18). The sample size was determined on the basis of sample sizes in proof-of-concept trials of DCS for anxiety disorders.^18, 21^ One participant was terminated from participation when it was determined that he was legally restricted from being on the university campus; four participants were lost to follow-up (DCS=1, PBO=3); and two participants missed one of the critical medication plus extinction sessions (DCS=1, PBO=1), resulting in a final sample of 30 (DCS, *n*=16; PBO, *n*=14), described in Table 1. A small number of individuals received an alcohol abuse diagnosis (*n*=3), preventing examination of differential effects by diagnosis. Of the 30 individuals completing treatment, two were unavailable for the 3-week follow-up session (DCS=2, PBO=0) and three individuals could not be reached for the telephone debriefing interview (DCS=1, PBO=2).

### Study protocol

All procedures were approved by the appropriate institutional review board and are described in Table 2. Although the study was principally designed to examine the effects of DCS on extinction of cue-elicited craving, it was nonetheless registered at clinicaltrials.gov (NCT01362309). For all visits, participants were required to be sober, verified via breath alcohol (Alco-Sensor IV, Intoximeters, St Louis, MO, USA). Initial eligibility was determined via telephone screen and an in-person screen. The in-person screen included a cue exposure protocol to determine the presence of cue reactivity. Following the in-person screen, eligible participants had blood drawn for a standard metabolic panel and assessment of recent drug use. The study physician (FTJ) conducted a general medical exam and reviewed the metabolic panel to determine medical eligibility.

Enrolled participants received four sessions of manualized motivational enhancement therapy over a 2-week period, adapted from the four-session Project MATCH treatment protocol.^46^ The extinction protocol was directly integrated into the treatment protocol and was presented as ‘self-control training.' Participants were given a clinical rationale akin to previous trials of CET: that environmental triggers are known to elicit cravings, that cue-elicited craving dissipates over time, and that extended exposure to these triggers is intended both to personally experience the dissipation of craving and to make the individual to become less reactive to triggers in their daily life. The treatment providers were MS-level clinicians (SFM, LRF, MKS and CMM) who received formal motivational interviewing training and were supervised by a licensed clinical psychologist (JM). The treatment was provided in a psychology department outpatient clinic and cue exposure/extinction sessions were completed in the same building in a naturalistic bar laboratory environment. Treatment sessions were video recorded and coded for therapist adherence to the clinical protocol (see below).

Sessions 1 and 3 included the medication/PBO administration and extinction procedures, and took place 1 week apart to minimize the probability of NMDA receptor desensitization. In these sessions, participants first received the motivational enhancement therapy sessions in the clinic and then proceeded to a neutral laboratory room. There, they received 50 mg DCS or PBO and waited 1 h for medication absorption. Medication administration was double-blind, with neither experimenters nor participants having any information about medication status. The active dose of 50 mg was selected on the basis of previous studies revealing significant effects on enhancement of fear extinction.^18, 21^ Both active medication and PBO were procured from a custom pharmacy (Pharmaceutical Specialties; Bogart, GA, USA) and were compounded with inert filler and 200 mg riboflavin to permit verification of consumption via urine fluorescence.^47^ Active medication and PBO capsules were identical in all features. Participants were required to not eat for 1 h before these sessions and had no access to food during the therapy sessions, meaning that the minimum duration between the last meal and the extinction protocol was either 3.5 h (session 1) or 3 h (session 3). To avoid discomfort from hunger, participants were provided with a meal during the post-extinction period. For smokers, smoking breaks were provided between the major units of the extended protocol, but not during aspects that were intended to have continuity (for example, after motivational enhancement therapy, but not during the extinction procedure).

Following the 1 h absorption period, participants underwent the 70-min extinction procedure, with intermittent assessment over the course of the session (Table 2). This comprised acute exposure to neutral cues (water) in a neutral laboratory room followed by repeated acute exposures to alcohol cues (the participant's preferred alcoholic beverage), in both cases consisting of visual, tactile, olfactory and proprioceptive stimuli (that is, picking up the beverage, holding it to one's nose, and taking deep inhalations of the smell of the beverage on repeated occasions). The alcohol portion involved five acute exposures and five passive exposure periods in alternating order (Table 2). That is, participants experienced five sequences of actively interacting with their preferred alcoholic beverage and then passively observing the beverage. All intra-lab cue exposure and extinction procedures were audiotaped for standardization. Additional procedural aspects of the medication administration are provided in supplementary materials.

Session 2 occurred between the two medication administrations, ~4 days following the first session, and was restricted to motivational enhancement therapy only. The clinical goal of the session was to follow up on the participants' motivation and progress after the extended first session. No cue exposure or extinction procedures were used because it was too soon to administer the medication again. The fourth session occurred ~4 days after the third session to provide timely follow-up on the participants' motivation and progress. A standard cue reactivity procedure (that is, neutral cues followed by alcohol cues) was used in the fourth session to assess changes in cue-elicited craving. Finally, a post-treatment follow-up assessment was completed 3 weeks later and also included a cue reactivity assessment. The follow-up cue exposure assessments were to assess the persistence of medication effects on cue-elicited craving.

### Assessment

As the primary dependent variable, craving was assessed using a best-practices approach, defining craving on a continuum of urges/desires^48, 49^ and using a multiple items.^50^ The items were: ‘*How much do you want to drink alcohol?,*' ‘*How much do you crave alcohol?,' ‘How much do you desire alcohol?*' and ‘*How high is your urge for alcohol?*' and have been successfully used in previous cue reactivity studies.^51, 52^ Craving was assessed following all acute beverage exposures and passive extinction periods. Cronbach's *α*s across all time points were >0.90. For ease of interpretation, percentage of scale maximum was used in all analyses.

Alcohol use disorder diagnosis was determined via the Structured Clinical Interview for DSM-IV Axis I Disorders (SCID).^53^ The SCID was administered by trained Master's-level clinicians and diagnoses were determined via case review by the study clinicians and the supervising licensed clinical psychologist (JM). A Timeline Followback^54^ was used to determine both the drinks per week eligibility criteria (past 28 days) and alcohol consumption during the study. The post-enrollment time windows were from the first session to the last and from the last session to the 3-week follow-up. Acute withdrawal symptoms were assessed for eligibility and at subsequent visits via the Clinical Withdrawal Assessment for Alcohol–Revised.^55^ Side effects were assessed using the SAFTEE (Systematic Assessment of Treatment Emergent Events).^56^ Participants completed the SAFTEE at the conclusion of each medication session and, after session 1, participants completed the SAFTEE at the beginning of each treatment session to report on any side effects during the interim period. As a further measure of tolerability, participants were asked whether they would participate in the study again and whether they would recommend participation to friends during debriefing. Process coding for the treatment sessions was completed via a checklist of 28 dichotomous items was used to code the presence of specific elements during the first session, which was the most structured. In addition, the GROMIT (Global Rating of Motivational Interviewing Therapist)^57^ was used to assess whether the therapist were consistent with the spirit of motivational interviewing for randomly selected subsequent sessions. Coding was conducted by study therapists, but no therapists coded their own sessions.

### Data analysis

Potential differences between the two conditions were examined using independent samples *t*-tests and *χ*^2^ tests for general characteristics, and a 2 (medication: DCS/PBO) × 2 (cue type: neutral/alcohol cues) mixed analysis of variance to assess any differences in initial cue reactivity. The primary analyses compared the effects of DCS versus PBO on subjective craving for alcohol via 2 (medication: DCS/PBO) × 2 (cue type: neutral cues/alcohol cues) mixed analyses of variance across the three bar laboratory exposures occurring during treatment and one exposure at the 3-week follow-up. Main effects and the interaction effect were examined to determine whether DCS affected craving in general and cue-elicited craving in particular. In addition, in the extinction sessions, we tested for the main effects of DCS versus PBO on craving via 2 (medication: DCS/PBO) × 2 (extinction: post first alcohol cues/post last alcohol cues) mixed analyses of variance, examining both main effects and the interaction. This permitted examining whether DCS affected the attenuation of craving over the course of extinction. To examine effects of DCS on drinking, we conducted one-way two-group analyses of covariance, covarying baseline drinking, on drinking immediately following treatment and at the 3-week follow-up. The continuous drinking variables were drinks per day, percent drinking days and heavy drinking days. No missing data were present for the primary outcome points and very rare for the follow-ups (see Participants); no data were imputed. Tolerability was examined via frequencies of symptoms on the SAFTEE, with *χ*^2^ tests to determine DCS/PBO differences.

## Results

### Preliminary analyses

No significant baseline differences were present between the two groups (Table 1). During the cue reactivity screening, a significant effect of cue type was present (that is, changes from neutral to alcohol cues; F(1,28)=61.29, *P*<0.001), reflecting an increase in craving; however, no medication condition effect was present (F(1,28)=0.03, *P*=0.86) and no cue × medication interaction was present (F(1,28)=0.003, *P*=0.96). Thus, at the start of the study, participants exhibited robust alcohol cue reactivity, but the two groups did not differ in the extent of this effect.

Process coding that follows pertains to the primary sample (that is, participants who received both doses, *n*=30). For the first session adherence, 87% of sessions were coded and the median number of elements completed was 26 of 28 (93%), suggesting high adherence. A total of 64 sessions were coded with the GROMIT and the mean percent of scale maximum was high across sessions: session 1—94% session 2—93% session 3—94% session 4—94%. In addition, the overall mean GROMIT score was similarly high, 94%. This suggests that the content of the intervention conformed to the spirit of motivational interviewing. In addition, all participants were observed consuming the pill and urine samples collected during the post-extinction period were observed to fluoresce, confirming that the medication was successfully administered.

At the conclusion of the study, 27 of the 30 participants completed the debriefing assessment. There were no differences between groups in guessing medication or PBO condition (*χ*^2^=0.06, *P*=0.97): ‘*Placebo*,' (DCS=60%, PBO=58%); ‘*Active Medication,*' DCS=27%, PBO=25% ‘*Unsure,*' DCS=13%, PBO=17%.

### Effects of DCS on alcohol cue reactivity and extinction

**Figure 1:**
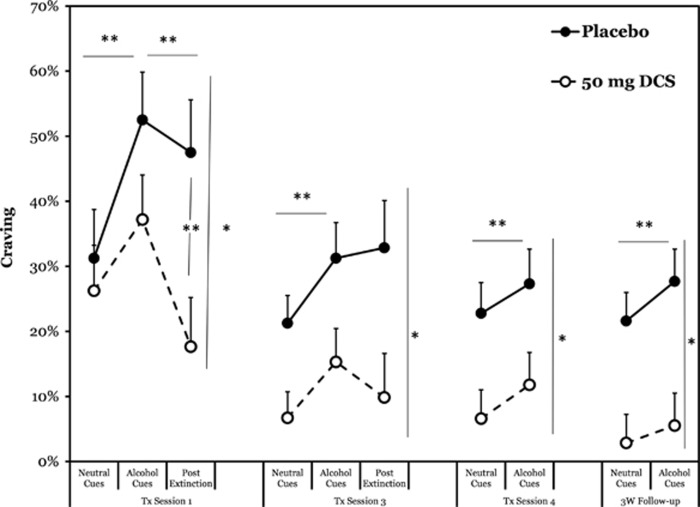
Effects of D-cycloserine (DCS) and placebo on craving for alcohol during cue reactivity and extinction across the study protocol. The DCS and placebo were administered 60 min before the cue exposure and extinction protocols in treatment (Tx) sessions 1 and 3. The initial cue exposure protocol lasted 14 min, including assessments; the extinction protocol comprised 63 min of subsequent active and passive exposure to personalized alcohol cues. Notations: horizontal bars with asterisks reflect significant within-subjects (time) main effects for a given interval; vertical bars with asterisks on the right reflect between-subjects (medication) main effects for the preceding epoch (including both epoch during session 3); vertical lines with asterisks between two time points reflect a significant difference in *post hoc* decomposition of an interaction effect; ^*^P⩽0.05, ^**^P⩽0.01.

During the first medication session, a significant effect of cue type was present (F(1,28)=9.33, *P*⩽0.01, *η*_p_^2^=0.25), reflecting a significant general increase in craving from neutral to alcohol cues; however, a significant medication effect was not observed (F(1,28)=1.37, *P*=0.25), nor was there a cue × medication interaction (F(1,28)=0.96, *P*=0.34, *η*_p_^2^=0.03). During the extended extinction period, there was a significant effect of extinction (F(1,28)=11.53, *P*⩽0.01, *η*_p_^2^=0.29), reflecting a decrease in craving across groups; a significant effect of medication (F(1,28)=5.15, *P*⩽0.05, *η*_p_^2^=0.16), reflecting lower overall levels of craving for individuals in the DCS condition; and a significant extinction × medication interaction (F(1,28)=4.04, *P*⩽0.05, *η*_p_^2^=0.13), reflecting an amplification of extinction effects in the DCS condition. Follow-up *post hoc* *t*-tests confirmed no significant difference between groups immediately following alcohol cues, but a significant difference at the end of extinction. Means, standard errors and statistical significance are presented in Figure 1.

At session 3, the second medication administration, there was a significant effect of cue type (F(1,28)=19.94, *P*⩽0.01, *η*_p_^2^=0.42), indicating increased craving from neutral to alcohol cues across groups, and a significant medication effect (F(1,28)=5.65, *P*⩽0.05, *η*_p_^2^=0.17), indicating lower levels of craving in the DCS condition compared with the PBO condition. The interaction effect was not significant (F(1,28)=0.11, *P*=0.74), suggesting similar increases in craving following the presentation of alcohol cues in both groups. During the extinction period, the DCS condition exhibited generally lower craving (F(1,28)=5.87, *P*⩽0.05, *η*_p_^2^=0.17), but neither extinction (F(1,28)=0.29, *P*=0.60), nor the extinction × DCS interaction (F(1,28)=0.97, *P*=0.33) were significant, suggesting that neither condition exhibited additional extinction and that the DCS did not exert differential effects. Means, standard errors and statistical significance are in Figure 1.

At session 4, the results revealed a significant effect of cue type (F(1,26)=9.07, *P*⩽0.01, *η*_p_^2^=0.25), reflecting increases in craving in response to alcohol cues. The medication effect was also significant (F(1,26)=5.60, *P*⩽0.05, *η*_p_^2^=0.17), revealing lower craving in the DCS condition at both time points, but the interaction was not significant (F(1,26)=0.04, *P*=0.85). Means, standard errors and statistical significance are in Figure 1.

At the 3-week follow-up, parallel patterns were observed, with significant effects of cue type (F(1,26)=7.33, *P*⩽0.01, *η*_p_^2^=0.22) and medication (F(1,26)=10.13, *P*⩽0.05, *η*_p_^2^=0.28), but no interaction effect (F(1,26)=1.10, *P*=0.30). Means, standard errors and statistical significance are in Figure 1.

### Effects on drinking and DCS tolerability

**Figure 2:**
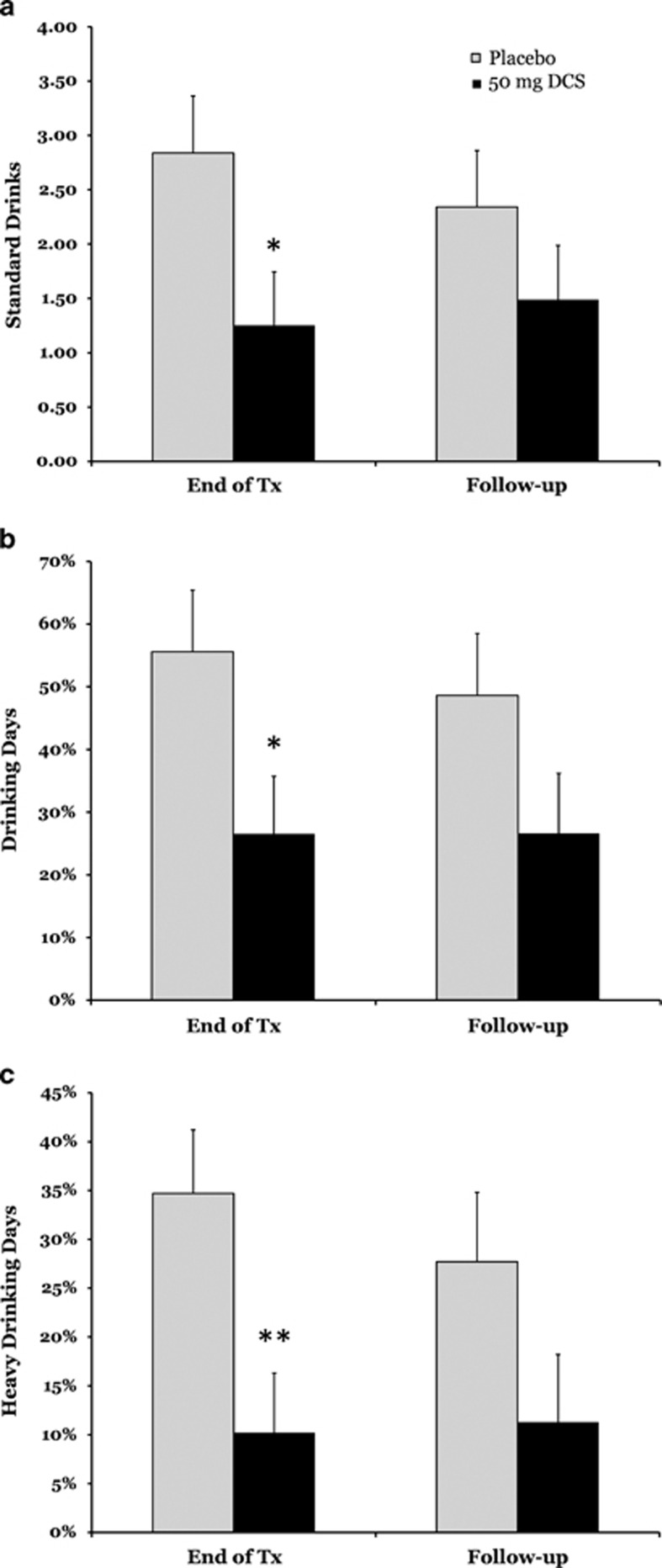
Effects of D-cycloserine (DCS) on short-term drinking outcomes at the conclusion of treatment (Tx)and 3-week follow-up. (**a**) Presents drinks per day, (**b**) presents percent drinking days and (**c**) presents percent heavy drinking days. Baseline drinking is covaried in all the analyses. ^*^P⩽0.05, ^**^P⩽0.01.

At the conclusion of treatment, participants who received DCS reported significantly fewer drinks per day (*P*⩽0.05), percent drinking days (*P*⩽0.05) and percent heavy drinking days (*P*⩽0.01). However, none of the medication effects were statistically significant at 3-week follow-up (*P*=0.25–0.62). These outcomes are depicted in Figure 2.

Active medication was associated with minimal side effects. The only notably higher side effect for DCS was drowsiness following the second administration, which was not statistically significant (*χ*^2^=2.71, *P*=0.10). Side effect frequencies are reported in Table 3. A large majority of participants reported that they would participate in the study again (89% yes, 4% not sure, 7% no) and would recommend it to their friends (96% yes, 4% not sure). The participant who would not participate again was in the PBO condition.

## Discussion

The current study investigated whether two administrations of 50 mg DCS, provided in the context of treatment for individuals with AUDs, would enhance extinction of cue-elicited alcohol craving. A significant DCS interaction was found during the first extinction session, with individuals in the active medication condition exhibiting steeper decreases in craving during the extinction period, and those group differences in craving persisted to the end of treatment and to a 3-week follow-up. Indeed, at the 3-week follow-up, participants in the DCS condition still exhibited very low absolute levels of craving before and following exposure to alcohol cues. A second dose of DCS was not found to differentially enhance extinction to alcohol cues significantly. This suggests it may not have been necessary, although it is also possible that it sustained the initial interaction effect. In addition, compared with PBO, participants in the DCS condition drank less while in treatment, although these differences were no longer significant at the follow-up. Across these findings, the DCS side effect profile was negligible, suggesting that the medication was well tolerated by individuals with AUDs.

These findings suggest that pharmacological enhancement of glutamatergic activity via the NMDA receptor increased the extinction of cue-elicited craving for alcohol. These results are in contrast to some previous human trials and number of factors need to be considered in their interpretation. First, the current study used a relatively small dose of DCS (50) when compared with the two previous studies, which used 125 mg^28^ and 250 mg.^30^ Larger doses might have caused DCS to act as an NMDA antagonist, leading to inhibition of extinction learning, and may even have had psychoactive alcohol-like effects.^58^ Second, the current study used a 1-week interval between extinction sessions, whereas one of the previous studies used a minimum of 2 days,^28^ which might have resulted in DCS having antagonist properties.^37, 38^ Third, this study required participants to exhibit cue-elicited craving as an eligibility criterion. Importantly, none of the previous studies established cue reactivity in their participants in advance of enrollment. Therefore, some of the participants in previous studies may not have exhibited increases in craving that were sufficiently large enough to be subsequently extinguished. Indeed, Watson *et al.*^30^ reported that approximately half of their sample exhibited no reactivity to the alcohol cues. This is particularly important because the level of initial cue-elicited craving fundamentally determines how much extinction of that craving is observable. Furthermore, the sample sizes in all of the studies to date have been relatively small and only a few non-reactors could substantially affect the findings.

It was also notable that the current study used a treatment-seeking clinical sample who received the medication and extinction in the context of outpatient treatment. Two of the previous studies used analogue samples of non-treatment-seeking heavy drinkers^28, 29^ and one previous study used an aftercare clinical sample but the experimental procedures were not framed as part of treatment.^30^ In this way, the current study is most similar to the study by Santa Ana *et al.*,^59^ which found 50 mg DCS significantly enhanced extinction among treatment-seeking nicotine-dependent outpatients. Thus, it appears that DCS enhances extinction to alcohol and tobacco cues when administered to clinical samples in the context of treatment according to specific dosing and administration parameters. This is also consistent with the anxiety disorders literature, suggesting that DCS enhances extinction in clinical samples seeking treatment,^18, 20, 21, 22^ but not analogue samples using *de novo* anxiety paradigms,^41, 42^ and only when the exposure procedures are successful.^60, 61^ The reason for significant effects in clinical sample may be due to the range of responses to environmental cues. Clinical severity is significantly associated with cue reactivity^62^ and thus clinical patients are more likely to exhibit robust cue-elicited craving. This, in turn, determines both how much extinction can be observed and the extent to which it can be enhanced.

More speculatively, it is possible that positive effects are observed when DCS is administered as part of treatment protocols for other reasons. It may be that the new learning that is taking place in the context of treatment is not simply the attenuation of the associative memory structure, but enhancement of other declarative forms of learning. For example, patients may experience enhancement of the strategies used for coping with cravings or may more deeply learn self-efficacy for experiencing cravings and not drinking. The current study cannot address these possibilities, but future studies should try to disentangle DCS effects on lower-order and higher-order learning processes.

It is important to consider these findings in the context of the study's strengths and weaknesses. With regard to strengths, the study emphasized high internal validity and met the recommended parameters of investigating DCS to enhance extinction. However, although the study was one of the largest on DCS and cue-elicited craving for alcohol, it still was relatively small in absolute size. In addition, as a proof-of-concept study on *in vivo* craving and extinction, the follow-up period was relatively short, only two administrations were used, and only one dose of DCS was used. Although conventional notions of reaching steady state drug levels do not apply to DCS, future studies should particularly examine a protracted administration schedule to observe maximal learning enhancement. Nonetheless, although the study was not designed or powered as a formal test of the medication's clinical efficacy, the significant positive short-term effects on drinking in this small sample suggest that positive long-term effects may be present in an appropriately powered clinical trial with a larger number of doses. Ideally, future studies will combine the putative mechanisms discussed above with clinical outcome measures to both address efficacy and, if present, clarify the operative underlying learning mechanisms. It is also worth noting that even though very high internal validity, including a sample with no psychiatric comorbidities whatsoever, is desirable in an early-stage proof-of-concept study, the current study permitted smokers as participants. However, this was based on the very high prevalence of smoking among individuals with AUDs^63^ and excluding smokers was considered too significant a threat to the external validity of the study. Whether smoking status is systematically related to DCS outcomes will be worth exploring in future studies.

More broadly, an important consideration is the role of context specificity in extinguishing cue-elicited craving. There is extensive experimental evidence that extinction is not ‘unlearning,' but new learning about the context dependency of environmental contingencies.^64^ For example, in animal models, it has been robustly demonstrated that for initial associative conditioning in one context, when extinction takes place in a different context, it does not generalize back to the first. Applied to extinction-based clinical interventions, context specificity is believed to substantially undermine the generalizability of in-session extinction to a patient's day-to-day environment and is putatively responsible for the modest clinical benefits of CET to date. Thus, even if DCS enhances extinction in treatment, it is not clear if it will enhance the generalizability of extinction beyond the treatment setting, potentially limiting its real-world clinical utility.

Despite this, there are some reasons to think DCS has clinical promise. To start, the current results revealed positive short-term benefits with two administrations in a relatively small sample. In addition, as noted earlier, trials of CET for alcohol dependence have reported modest positive outcomes,^15^ suggesting there is a baseline positive signal that is viable for enhancement and maximization. Similarly, human laboratory studies of cue-elicited craving in multiple contexts suggest that substantial generalizability does in fact take place^43, 52^ and again would be the target of maximization by DCS. Finally, it is possible that DCS enhances the declarative learning processes discussed above and those higher-order relationships would be expected to generalize beyond the immediate treatment context. However, we recognize that this is conjecture. Alternatively, it is also possible that the current findings may only contribute to understanding the neurobiology of cue-elicited craving and that, because of the context dependency of extinction learning, DCS may have limited clinical utility. This is, of course, fundamentally an empirical question and one that needs to be addressed in future clinical investigations. The current results provide a strong basis for pursuing these questions further.

## Figures and Tables

**Table 1:** Participant characteristics

*Variable*	*%/Mean (s.d.)/Median (IQR)*		P
	*DCS (*n*=16)*	*PBO (*n*=14)*	
Sex	63% Male	43% Male	0.28
Age	41.88 (14.90)	42.79 (13)	0.86
Education (Years)	14.69 (2.68)	15.71 (2.37)	0.28
Income ($)	20 000–29 999 (0–9999 to >80 000)	30 000–39 999 (10 000–19 999 to 50 000–59 999)	0.83
Race	81% White; 13% Black; 6% Mixed Race	86% White; 14% Black	0.63
Hispanic ethnicity	6%	0%	0.34
% Drinking days	83.26% (14.97)	84.18% (21.09)	0.89
% Heavy drinking days	62.28% (28.81)	70.15% (28.14)	0.46
Drinks per day	5.317 (2.81)	7.870 (6.31)	0.16
AUD symptom count	5.06 (2.05)	5.86 (1.96)	0.29
Abuse/dependence	6%/94%	14%/86%	0.46
Smoker	50%	64%	0.45

Abbreviations: AUD, alcohol use disorder; DCS, D-cycloserine; IQR, interquartile range; PBO, placebo.

Continuous variables were examined using *t*-tests, categorical variables were examined using *χ*^2^ tests; heavy drinking days indicate consuming 5/4 drinks in a given day for males/females. Time frame for drinking refers to the last 28 days; time frame for AUD symptoms refers to the last year.

**Table 2:** General study protocol and alcohol cue reactivity and extinction protocol

*Day*	*Procedure*	*Duration (Min)*
*General protocol*		
−4	Telephone screen	10
−3	In-person screen	120
−2	Blood draw (metabolic panel and drug screen)	60
−1	Medical screen	60
1	Treatment session #1: MET+EXT+DCS/PBO	240
5	Treatment session #2: MET	60
8	Treatment session #3: MET+EXT+DCS/PBO	240
12	Treatment session #4: MET+CR	60
33	Three-week follow-up: CR	60
		
*Extinction protocol (days 1 and 8)*		
	Neutral cue exposure^a^	7
	Acute alcohol cue exposure^b^	7
	Passive alcohol cue exposure^c^	7
	Acute alcohol cue exposure	7
	Passive alcohol cue exposure	7
	Acute alcohol cue exposure	7
	Passive alcohol cue exposure	7
	Acute alcohol cue exposure	7
	Passive alcohol cue exposure	7
	Acute alcohol cue exposure	7
	Passive alcohol cue exposure	7

Abbreviations: CR, acute alcohol cue exposure; within the extinction protocol, note that all cue exposure and extinction periods were followed by craving assessments; DCS/PBO, D-cycloserine/placebo; EXT, extinction (that is, prolonged exposure to alcohol cues with response prevention); MET, motivational enhancement therapy.

a Direct interaction with water cues.

b Direct interaction with participant's preferred alcoholic beverage.

c Passive observation of participant's preferred alcoholic beverage.

**Table 3:** Tolerability of DCS and placebo

	*S1*		*S2*		*S3A*		*S3B*		*S4*	
	*PBO*	*DCS*	*PBO^aa^n=13.*	*DCS*	*PBO^a^*	*DCS*	*PBO*	*DCS*	*PBO^a^*	*DCS^bb^n=15.*
Drowsy (%)	21	19	8	13	0	6	7	31	15	0
Dizzy (%)	14	0	8	0	0	6	7	0	0	0
Headache (%)	0	0	23	6	8	0	0	0	15	0
Slurred speech (%)	0	0	0	0	0	0	0	0	0	0
Tingling (%)	0	0	0	0	0	0	0	0	0	0
Mental confusion (%)	0	0	0	13	8	0	0	0	0	0
Irritability (%)	0	0	31	13	8	0	0	0	15	0
Anxiety (%)	0	6	8	0	8	0	0	0	8	0
Skin rash (%)	0	0	0	6	0	6	0	0	0	0
Aggression (%)	0	0	15	0	0	0	0	0	0	0

Abbreviations: DCS, D-cycloserine; PBO, placebo; S1, 1 h following the first medication administration; S2, intermediate session, ~3 days after the first administration; S3A, ~7 days after first administration, S3B, 1 h following the second medication administration; S4, the fourth treatment session, ~1 week following the second medication administration.

Proportions represent participant endorsement of a symptom for a given time period. No significant differences in side effects were present between the two groups.

## Notes

RS is a member of the Advisory Board of D&A Pharma and has received travel expenses and honoraria from this company. He has received consulting fees from Transcept Pharmaceuticals and Pharmaceutico CT. The remaining authors declare no conflict of interest.
